# Supplementary material for: The Novel Structural Variation in the GHR Gene Is Associated with Growth Traits in Yaks (Bos grunniens)
Source: Animals (Basel). 2023 Feb 26;13(5):851. doi: 10.3390/ani13050851 (PMC10000137; doi:10.3390/ani13050851)
Supplement: Supplementary file 1 [file animals-13-00851-s001.zip › animals-2024265-Supplementary.pdf]

Supplementary Table S1. The overall average of the growth parameters

Age	Growth trait	Average	Maximum	Minimum	Standard Deviation	Coefficient of variation
6 months (n=315)	Body weight	84.18	117	58	10.30	12.24%
	Body length	94.47	108	82	5.19	5.50%
	Body height	91.95	116	73	7.40	8.05%
	Chest girth	123.99	144	100	7.82	6.31%
12 months (n=315)	Body weight	82.61	113	48	10.56	12.78%
	Body length	90.52	102	81	4.17	4.61%
	Body height	95.94	113	80	4.97	5.18%
	Chest girth	117.15	134	102	5.09	4.35%
18 months (n=226)	Body weight	122.39	168	76	13.01	10.63%
	Body length	101.88	124	80	6.02	5.91%
	Body height	101.64	120	85	5.79	5.69%
	Chest girth	138.33	182	107	10.15	7.34%
30 months (n=180)	Body weight	155.42	203	108	15.23	9.80%
	Body length	99.55	117	90	5.00	5.02%
	Body height	113.17	126	96	5.70	5.03%
	Chest girth	146.97	173	122	8.27	5.62%

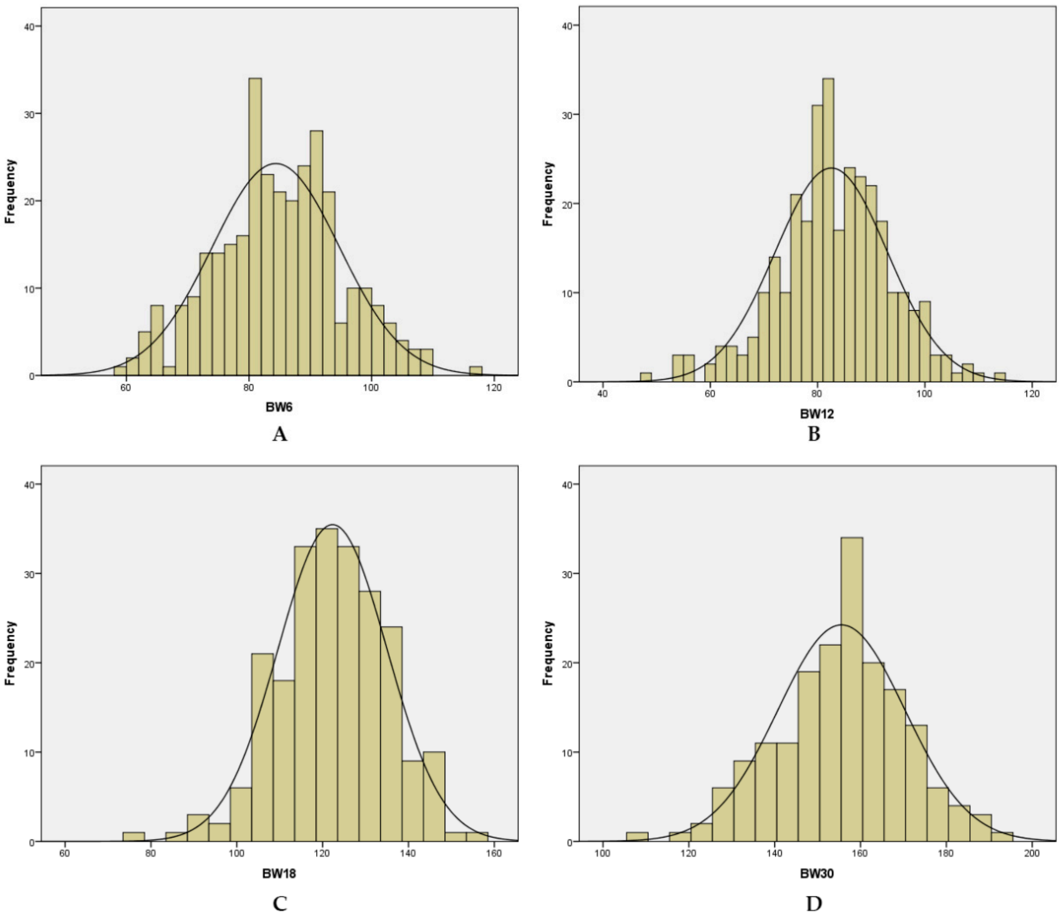

Figure S1: Histogram of normal distribution of body weight frequency of yaks at different ages.

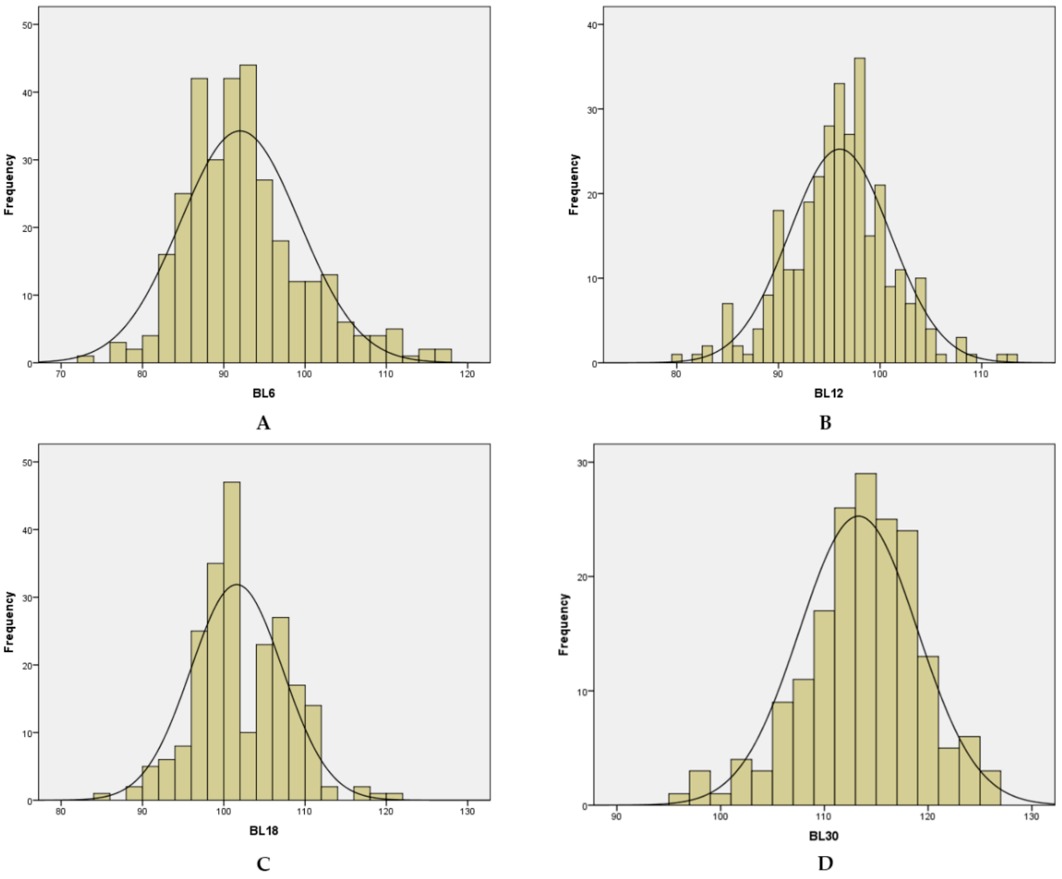

Figure S2: Histogram of normal distribution of yak body length frequency at different months.

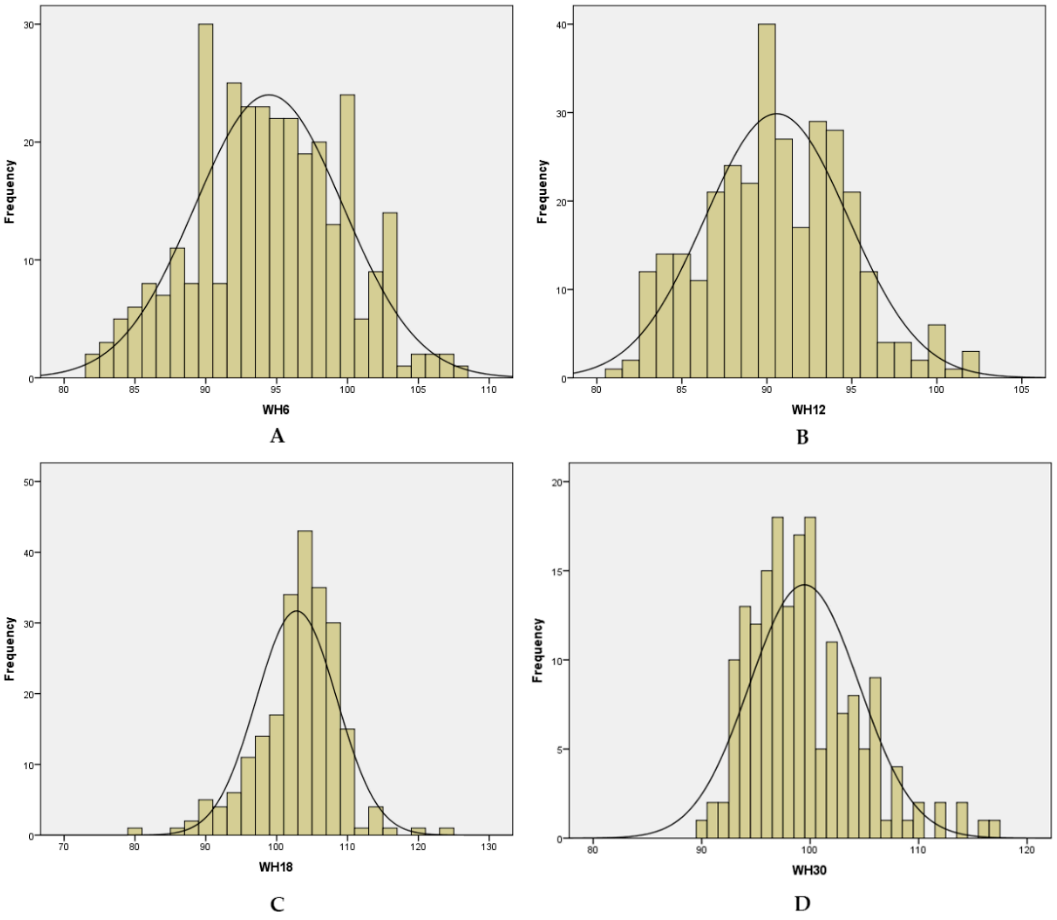

Figure S3: Histogram of normal distribution of yak body height frequency at different months.

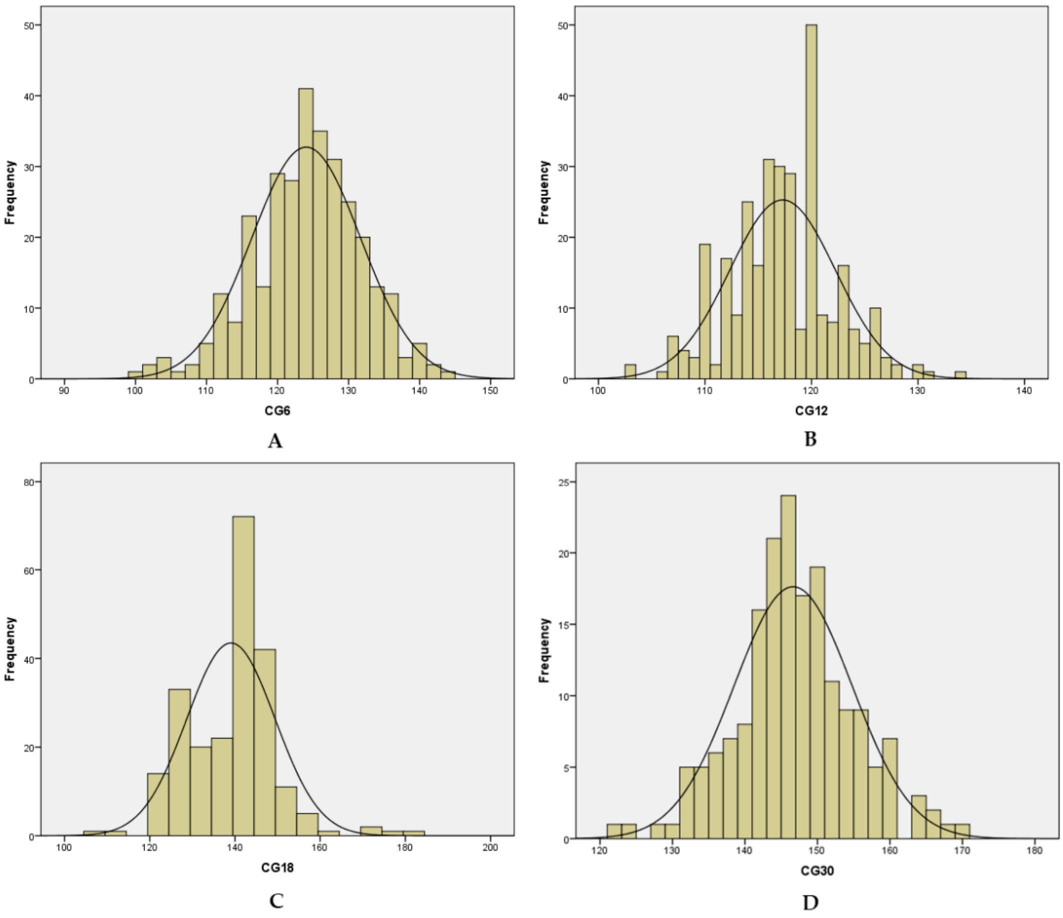

Figure S4: Histogram of normal distribution of chest girth frequency of yaks at different ages.
